# Shedding light on the performance of a pyrosequencing assay for drug-resistant tuberculosis diagnosis

**DOI:** 10.1186/s12879-016-1781-y

**Published:** 2016-08-31

**Authors:** Sophia B. Georghiou, Marva Seifert, Shou-Yean Lin, Donald Catanzaro, Richard S. Garfein, Roberta L. Jackson, Valeriu Crudu, Camilla Rodrigues, Thomas C. Victor, Antonino Catanzaro, Timothy C. Rodwell

**Affiliations:** 1 Department of Medicine, University of California San Diego, La Jolla, CA USA; 2 California Department of Public Health, Richmond, CA USA; 3 University of Arkansas, Fayetteville, AR USA; 4 Microbiology and Morphology Laboratory, Institute of Phthisiopneumology, Chisinau, Moldova; 5 Department of Microbiology, P.D. Hinduja Hospital and Medical Research Centre, Mumbai, India; 6 Division of Molecular Biology and Human Genetics, Stellenbosch University, Stellenbosch, South Africa

**Keywords:** Drug-resistant tuberculosis, Pyrosequencing, Molecular diagnostics, Performance evaluation

## Abstract

**Background:**

Rapid molecular diagnostics, with their ability to quickly identify genetic mutations associated with drug resistance in *Mycobacterium tuberculosis* clinical specimens, have great potential as tools to control multi- and extensively drug-resistant tuberculosis (M/XDR-TB). The Qiagen PyroMark Q96 ID system is a commercially available pyrosequencing (PSQ) platform that has been validated for rapid M/XDR-TB diagnosis. However, the details of the assay’s diagnostic and technical performance have yet to be thoroughly investigated in diverse clinical environments.

**Methods:**

This study evaluates the diagnostic performance of the PSQ assay for 1128 clinical specimens from patients from three areas of high TB burden. We report on the diagnostic performance of the PSQ assay between the three sites and identify variables associated with poor PSQ technical performance.

**Results:**

In India, the sensitivity of the PSQ assay ranged from 89 to 98 % for the detection of phenotypic resistance to isoniazid, rifampicin, fluoroquinolones, and the injectables. In Moldova, assay sensitivity ranged from 7 to 94 %, and in South Africa, assay sensitivity ranged from 71 to 92 %. Specificity was high (94–100 %) across all sites. The addition of *eis* promoter sequencing information greatly improved the sensitivity of kanamycin resistance detection in Moldova (7 % to 79 %). Nearly all (89.4 %) sequencing reactions conducted on smear-positive, culture-positive specimens and most (70.8 %) reactions conducted on smear-negative, culture-positive specimens yielded valid PSQ reads. An investigation into the variables influencing sequencing failures indicated smear negativity, culture negativity, site (Moldova), and sequencing of the *rpoB, gyrA*, and *rrs* genes were highly associated with poor PSQ technical performance (adj. OR > 2.0).

**Conclusions:**

This study has important implications for the global implementation of PSQ as a molecular TB diagnostic, as it demonstrates how regional factors may impact PSQ diagnostic performance, while underscoring potential gene targets for optimization to improve overall PSQ assay technical performance.

**Trial registration:**

ClinicalTrials.gov (#NCT02170441). Registered 12 June 2014.

**Electronic supplementary material:**

The online version of this article (doi:10.1186/s12879-016-1781-y) contains supplementary material, which is available to authorized users.

## Background

In 2014, 9.6 million new cases of tuberculosis (TB) and 1.5 million TB-associated deaths were reported worldwide [1]. Although the incidence of new TB cases has continued to fall over the past decade, the incidence of multi- and extensively drug-resistant TB (M/XDR-TB) has been stable, undermining TB eradication efforts. MDR-TB is defined as TB that has developed resistance to the first-line anti-tuberculosis drugs isoniazid (INH) and rifampicin (RIF). XDR-TB is MDR-TB that has developed additional resistance to the fluoroquinolones (FQs) and at least one of the injectable compounds [amikacin (AMK), kanamycin (KAN) and/or capreomycin (CAP)]. An estimated 480,000 people developed MDR-TB while 190,000 deaths were attributed to MDR-TB in 2014 [1]. Alarmingly, only 26 % of the estimated MDR-TB infections globally were detected in 2014 [1]. This means that over one third of a million people suffered from undiagnosed and untreated drug-resistant TB, which is a significant risk for high mortality and continued transmission of M/XDR-TB.

The conventional methodology for diagnosis of drug-resistant TB (DR-TB) has not changed for decades, and relies upon mycobacterial culture and drug susceptibility testing (DST) in solid or liquid media. These methods yield results only after weeks to months of cell culture, and require biosafety conditions that are complex and expensive to implement in low- and middle-income countries. MDR- and XDR-TB patients waiting for growth-based diagnostic test results before appropriate treatment is started remain contagious and at increased risk of death.

Rapid molecular diagnostics for M/XDR-TB have great potential to shorten the time to DR-TB diagnosis and appropriate treatment. Pyrosequencing (PSQ) is a real time, rapid method for sequencing fragments of genomic DNA. PSQ assays have been previously established as valid technologies to rapidly and accurately identify mutations associated with drug resistance in *Mycobacterium tuberculosis* (*Mtb*) isolates and in clinical specimens [2–8]. The commercially available Qiagen PyroMark Q96 PSQ platform has been validated as an M/XDR-TB diagnostic assay and is currently in use in by the Microbial Diseases Laboratory in the California Department of Public Health for rapid detection of M/XDR-TB in the United States, having been validated for clinical use [9, 10]. One study validating this PSQ assay against conventional methods in high burden settings found strong correlations with phenotypic DST, with sensitivity values ranging from 86 to 100 % and specificity values ranging from 99 to 100 % for all drugs tested [9]. The Global Consortium for Drug-resistant TB Diagnostics (GCDD) also conducted a large-scale, multisite study to evaluate the ability of this assay to accurately predict TB phenotypic drug resistance profiles, and found test performance to vary across diverse clinical environments. Overall assay sensitivity ranged from 50 to 95 %, and the number of interpretable results ranged from 73 to 88 % among *Mtb* culture-positive specimens [11]. These variations highlight potential limitations of the assay. This study examines the detailed diagnostic and technical performance of a PSQ assay for M/XDR-TB diagnosis in three diverse clinical sites and describes modifications that could improve overall diagnostic and technical performance of the PSQ assay.

## Methods

### Study population

Briefly, three diverse clinical sites (Chisinau, Moldova, Port Elizabeth, South Africa, and Mumbai, India) were selected for this study [12]. Newly-presenting TB patients over 5 years of age were eligible for the study if they were known to be acid-fast bacilli (AFB) smear-positive (defined as 1+ or greater within prior 14 days) or suspected of having active pulmonary TB and having one or more reason to be considered to have DR-TB, and provided informed consent for the study. Patients unable to provide 7.5 mL of sputum were excluded, along with subjects who had second-line DST in the prior three months. A total of 1128 patients meeting the above criteria were enrolled in the GCDD study from April 24, 2012 to June 27, 2013.

### Acid-Fast Bacilli (AFB) smear and drug-susceptibility testing

AFB smear testing was performed on all isolates, and smear grading was determined in the first 2 weeks following enrollment. All phenotypic drug susceptibility profiles were established using the Mycobacterial Growth Indicator Tube (MGIT) 960 platform. These MGIT DST results served as reference standard in our study. All specimens were tested for resistance to INH, RIF, two FQs [moxifloxacin (MOX) and ofloxacin (OFX)], and three injectable drugs (AMK, KAN and CAP) using standard manufacturer protocols [13] and previously-published and World Health Organization-recommended critical concentrations for MGIT-based DST [12, 14].

### DNA extraction, PCR and molecular targets

Crude DNA was extracted from each decontaminated, concentrated sputum (sediment) by heating the cell suspensions in a water bath at 100 °C [9, 12]. PCR master mixes were prepared and amplification reactions were carried out as previously reported [9]. Table 1 lists all primers used for PCR and sequencing reactions. Our PSQ assay included one reaction to identify *Mtb* and seven reactions to detect specific mutations in drug resistance-associated gene regions. The molecular target IS*6110* was considered confirmatory for identification of *Mtb*. However, since the marker is not 100 % reliable, especially for Indian strains of *Mtb* [15–19], we included findings for specimens deemed indeterminate for presence of the IS*6110* marker via PSQ as long as at least one other *Mtb* gene yielded a sequencing result. This practice is in accordance with similar PSQ studies as, apart from the *rrs*, the primers utilized in sequencing reactions are highly specific for *Mtb* [9]. A negative H_2_O control was used for every target in each run.

**Table 1:** Primers Utilized in PCR and Pyrosequencing (PSQ) Reactions

Genes	Target	Forward primer	Reverse primer	Sequencing primer	Detection range	Reference
Mtb Identification	IS*6110*	Biotin-CCGCCAACTACGGTGTTTA	CAGGCCGAGTTTGGTCAT	GGCCACCTCGATGCC	Multiple	[9]
Isoniazid-R	*katG*	Biotin-CGGAACCGGTAAGGACGC	CCATTTCGTCGGGGTGTTC	TCCATACGACCTCGAT	Codons 312 to 316	[9]
	*inhA*	Biotin-ACGCTCGTGGACATACCG	CAGTGGCTGTGGCAGTCA	TGTGGCAGTCACCCC	Position −4 to −20	[9]
	*ahpC*	TCCTCATCATCAAAGCGGACAAT	Biotin-CGATGCCGATAAATATGGTGTGAT	CATTTGGTTGCGACAT	Position −4 to −23	[9]
Rifampin-R	*rpoB1*	GGAGGCGATCACACCGCAGACGTT	Biotin-CCTCCAGCCCGGCACGCTCACGT	GCGATCAAGGAGTTCTTC	Codons 507 to 521	[6, 9]
	*rpoB2*	TTTCGATCACACCGCAGACGTT	Biotin-AAAGGCACGCTCACGTGACAGAC	CAGAACAACCCGCTG	Codons 522 to 533	[6, 9]
Fluoroquinolone-R	*gyrA*	AATGTTCGATTCCGGCTTCC	Biotin-CGGGCTTCGGTGTACCTCAT	CAACTACCACCCGCAC	Codons 88 to 95	[2]
Injectable-R	*rrs*	TAAAGCCGGTCTCAGTTCGGAA^a^C	Biotin-CAGCTCCCTCCCGAGGGTTA	CTTGTACACACCGCC	Position 1397 to 1406	[9]
Kanamycin-R	*eis*	Biotin-GGCTACACAGGGTCACAGTC	GCCAGACACTGTCGTCGTAATATTC	CAGACACTGTCGTCG	Position −5 to −47	This study

^a^“A” was substituted for “T” in the natural sequence to improve specificity

*Mtb Mycobacterium tuberculosis, −R* -resistant

### Pyrosequencing (PSQ)

We used the PyroMark Q96 ID system (Qiagen, Valencia, CA) to perform PSQ on specific regions of the *ahpC* and *inhA* promoters and the *katG*, *rpoB*, *gyrA*, and *rrs* genes, sequencing two different parts of *rpoB* in two separate reactions, as described previously [9]. Sequencing of these targets was completed at the respective clinical sites. Sequenced gene regions are outlined in Table 1. Variants relative to the *Mtb* H37Rv reference strain (ATCC 27294) were identified automatically from generated PSQ pyrograms using IdentiFire software (Qiagen, Valencia, CA). All samples that did not provide PSQ queries with a 100 % match to library wildtype or mutant sequences were repeated in duplicate. Samples that still did not provide confirmatory sequence and samples for which contradictory hits were obtained for any given target were deemed genotypically indeterminate.

Upon completion of the study, *eis* promoter sequencing capabilities were added to the platform by designing primers specific for sequencing the *eis* promoter of *Mtb* (Table 1), and updating the system’s library for query read identification via the IdentiFire software. *eis* sequencing reactions used PCR and PSQ parameters identical to the other assay targets. In sequencing the *eis* promoter, DNA extracted from specimens from India were sequenced on-site, while DNA extracted from specimens from Moldova and South Africa were sequenced using a PyroMark Q96 ID system at the University of California, San Diego. As for the other targets, all *eis* queries that did not 100 % match reference library sequences were repeated in duplicate.

### Pyrosequencing (PSQ) diagnostic performance

In order to comment on the validity of the PyroMark PSQ platform in establishing *Mtb* drug resistance profiles, we calculated sensitivity and specificity for each drug by comparing PyroMark findings to conventional MGIT phenotypic DST results in each clinical site. Only those specimens with both sequencing results and DST results for the relevant drugs of interest were included in diagnostic performance estimate calculations. Sensitivity was calculated as the number of phenotypically resistant specimens in which a resistance-associated mutation was found via PSQ, divided by the number of phenotypically resistant specimens. Specificity was calculated as the number of phenotypically susceptible specimens in which no resistance-associated mutation was found via PSQ, divided by the number of phenotypically susceptible specimens. INH resistance was determined with PSQ via the presence of known resistance-conferring mutations in at least one of three genes (*inhA*, *katG* and *ahpC*), RIF resistance through presence of at least one resistance-associated mutation in one of two *rpoB* gene regions, injectable resistance through the presence of the 1401G or 1402T mutation in the *rrs* gene, and FQ resistance via the presence of resistance-conferring mutations in the *gyrA* gene. KAN resistance was determined via the presence of the 1401G or 1402T mutation in the *rrs* gene or the presence of a resistance-associated mutation in the *eis* promoter. Confidence intervals for sensitivity and specificity of individual mutations were determined using the score/efficient score method with continuity correction [20, 21]. Diagnostic performance differences were noted between the sites based upon the presence of non-overlapping confidence intervals for sensitivity or specificity calculations for any particular drug.

### Analysis of pyrosequencing (PSQ) technical performance

Sequencing success was first determined for smear- and culture-negative and positive samples by calculating the proportion of the total PSQ reactions conducted for those samples that yielded interpretable sequencing results. The variables associated with poor PSQ technical performance, defined as the inability to obtain interpretable sequencing results, were then investigated by logistic regression, using STATA 13.1 Software (StataCorp, College Station, TX, USA). The outcome variable was an “indeterminate” result, or the inability to obtain a PSQ read that generated a 100 % match with a target library sequence. Covariates evaluated included: smear negativity, culture negativity, clinical site and gene target. A bivariate analysis was first conducted to generate unadjusted odds ratios for each variable. Variables with *p*-value <0.20 were considered for inclusion in the final model. Covariates included in the final multivariate model with *p*-value <0.05 were considered significant.

In order to further comment on the underlying reasons for PSQ failures, all indeterminate results were categorized according to type of observed error. Categories included: no read error, homopolymer error, instrument error, mixed population, new mutation, or other error. No read errors were defined as unresolved errors where few or no peaks were seen in the resulting PSQ pyrograms in all sequencing reactions for a given gene target. Homopolymer errors were unresolved errors that occurred due to IdentiFire software mischaracterization of pyrogram peak height at one or more bases in any PSQ reaction for a given gene target. Instrument errors were unresolved errors resulting from incorrect instrument reagent dispensation or camera detection errors, where one or more peaks in the resulting pyrogram were seen as a split peak (two small peaks) below IdentiFire peak detection threshold. Mixed populations occurred when all three pyrograms obtained for any gene target were identical but did not match a confirmatory sequence in the sequencing library due to the presence of two peaks in a given mutation region- representing both wildtype and mutant sequences. New or novel mutations were confirmed when three unambiguous, identical pyrograms were obtained for any gene target but did not match a sequence in the reference sequence library of known wildtype and common mutations in that region. Finally, the other error category included all other errors, including unknown errors or a combination of error types that could not be attributed to a single source. All PSQ indeterminates were characterized according to one of these reasons for error, and the numbers of errors falling into the different categories were summarized for each gene target.

### Human research conduct

Our study was approved by the Institutional Review Board of the University of California, San Diego and by the Institutional Review Boards of the respective clinical sites.

## Results

### Culture and Drug Susceptibility Testing (DST) results

Of 1128 patients enrolled in the study, 914 (81 %) provided *Mtb* culture-positive pulmonary sputum samples. One of the remaining 214 samples was contaminated, and the rest were *Mtb* culture-negative. MGIT DST could not be performed, or did not yield results, for seven of the 914 culture-positive clinical specimens. One additional specimen did not yield a valid DST result for the evaluation of phenotypic MOX resistance. Four hundred fifty-four (40 %) of the 1128 patients enrolled in the study had MDR-TB and 80 (7 %) had XDR-TB, as determined by MGIT DST results. Thus, 907 results were available for this analysis (906 for MOX).

### Sensitivity and Specificity of Pyrosequencing (PSQ) as Compared to Phenotypic Testing

PSQ diagnostic performance for each TB treatment drug in each clinical site is detailed in Table 2.

**Table 2:** Pyrosequencing diagnostic performance by clinical site

	India (*n* = 492)			Moldova (*n* = 226)			South Africa (*n* = 196)		
	Sensitivity	Specificity	Agreement	Sensitivity	Specificity	Agreement	Sensitivity	Specificity	Agreement
INH	98 % (0.96–0.99)	97 % (0.90–1)	98 % (0.96–0.99)	94 % (0.88–0.97)	96 % (0.87–0.99)	95 % (0.90–0.97)	71 % (0.53–0.85)	94 % (0.87–0.98)	88 % (0.81–0.93)
RIF	98 % (0.95–0.99)	100 % (0.94–1)	98 % (0.96–0.99)	94 % (0.86–0.98)	100 % (0.92–1)	97 % (0.92–0.99)	77 % 0.54–0.91)	98 % (0.91–1)	94 % (0.87–0.97)
MOX	96 % (0.92–0.98)	96 % (0.92–0.98)	96 % (0.94–0.98)	67 % (0.39–0.87)	100 % (0.97–1)	97 % (0.92–0.99)	82 % (0.48–0.97)	99 % (0.96–1)	98 % (0.94–1)
OFX	96 % (0.93–0.98)	99 % (0.96–1)	97 % (0.95–0.99)	64 % (0.36–0.86)	99 % (0.96–1)	96 % (0.91–0.98)	90 % (0.54–0.99)	99 % (0.96–1)	99 % (0.95–1)
AMK	94 % (0.82–0.98)	100 % (0.98–1)	99 % (0.98–1)	33 % (0.11–0.65)	99 % (0.96–1)	95 % (0.91–0.98)	92 % (0.60–1)	98 % (0.94–0.99)	98 % (0.93–0.99)
KAN	89 % (0.76–0.95)	100 % (0.98–1)	99 % (0.97–0.99)	7 % (0.02–0.18)	99 % (0.95–1)	71 % (0.64–0.77)	92 % (0.60–1)	98 % (0.94–0.99)	97 % (0.94–0.99)
KAN (+*eis*)	93 % (0.81–0.98)	91 % (0.88–0.94)	91 % (0.88–0.94)	79 % (0.66–0.88)	95 % (0.90–0.98)	90 % (0.85–0.94)	92 % (0.60–1)	98 % (0.93–0.99)	97 % (0.93–0.99)
CAP	94 % (0.81–0.98)	99 % (0.98–1)	99 % (0.97–0.99)	40 % (0.14–0.73)	99 % (0.96–1)	96 % (0.92–0.98)	85 % (0.54–0.97)	98 % (0.94–0.99)	97 % (0.92–0.99)

*INH* isoniazid, *RIF* rifampicin, *MOX* moxifloxacin, *OFX* ofloxacin, *AMK* amikacin, *KAN* kanamycin, *CAP* capreomycin

No major differences in the specificity of the PSQ assay for the detection of resistance to any antibiotic were observed between the three sites, with assay specificity ranging from 94 to 100 % for all drugs in all sites prior to the addition of the *eis* promoter. The PSQ assay did, however, show differences in diagnostic sensitivity for various drugs between the three sites. For the detection of INH resistance, as seen in the presence of distinct 95 % confidence intervals, the assay demonstrated lower sensitivity in South Africa (71 %) than in either India (98 %) or Moldova (94 %). For the detection of RIF resistance, the assay demonstrated lower sensitivity in South Africa (77 %) than in India (98 %). For the detection of FQ resistance, the assay demonstrated lower sensitivity in Moldova (64–67 %) than in India (96 %). The sensitivities of the PSQ assay for the detection of resistance to the injectable drugs varied greatly between the three sites. The sensitivity of the assay for the detection of AMK resistance was 94 % in India, 33 % in Moldova, and 92 % in South Africa. The sensitivity for the detection of CAP resistance was 94 % in India, 40 % in Moldova, and 85 % in South Africa. The sensitivity of the assay for the detection of KAN resistance showed the greatest variation of all the injectables between the three sites: 89 % in India, 7 % in Moldova, and 92 % in South Africa. For the detection of injectable resistance, our PSQ assay demonstrated lower sensitivity in Moldova than in India for all drugs, though 95 % confidence intervals overlapped with South African estimates for all but KAN resistance detection. By far, the most notable difference in assay sensitivity between the three sites was for the detection of KAN resistance in Moldova, where only 7 % (95 % CI 0.02–0.18) of the 57 phenotypically KAN-resistant specimens were found to have the *rrs* 1401G mutation, compared to 89 % in India and 92 % in South Africa.

### KAN resistance upon *eis* promoter addition

Prior to the addition of *eis* promoter sequencing capabilities to the PyroMark platform, overall sensitivity of the PSQ assay was lowest for the overall detection of KAN resistance (50.4 %) [11]. The addition of *eis* promoter mutations as predictors of KAN resistance increased the sensitivity estimate to 85.8 %, but decreased overall specificity for KAN from 99.3 to 93.3 %. In India, the addition of the *eis* promoter region to the assay increased test sensitivity for KAN resistance from 89 to 93 %, but decreased test specificity from 100 to 91 %. The addition of the *eis* promoter greatly increased test sensitivity for KAN resistance in Moldova, from 7 to 79 %, but decreased test specificity from 99 to 95 %. In South Africa, test sensitivity remained unchanged upon the addition of *eis* promoter sequencing capabilities, as no *eis* promoter mutations were identified in South African specimens.

### Pyrosequencing (PSQ) success by smear- and culture-result

**Fig. 1:**
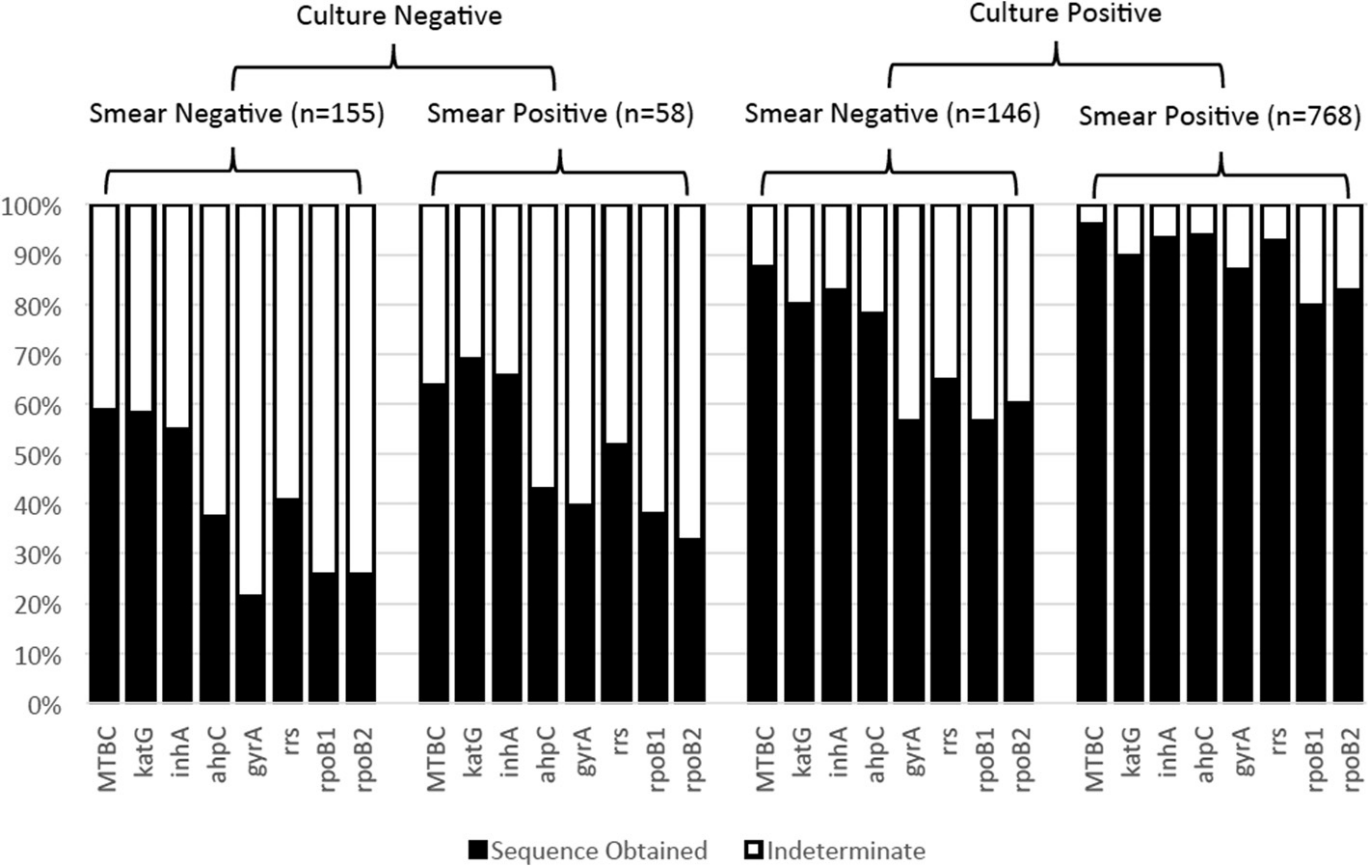
Pyrosequencing Technical Performance (Sequencing Success) by Acid-Fast Bacilli Smear and Culture Result

PSQ of the IS*6110*, *katG*, *inhA*, *ahpC*, *gyrA*, *rrs*, and two *rpoB* gene targets (regions outlined in Table 1) was performed on all samples, regardless of culture- and smear-status, at the respective clinical sites. Altogether, 9016 gene target regions were pyrosequenced between the three sites. Overall, 86.7 % of all smear-positive specimens and 86.4 % of all culture-positive specimens yielded valid PSQ reads, while 54.9 % of all smear-negative specimens and 43.1 % of all culture-negative specimens gave valid sequence reads for the given gene targets. Figure 1 summarizes PSQ reaction success for each target gene region, stratified by smear- and culture-result. The IS*6110* gene marker had the highest frequency of successful PSQ reactions for all reactions, at 88 %, followed by *inhA*, *katG*, *ahpC*, *rrs*, *gyrA*, *rpoB2*, and finally *rpoB1* at 85, 83, 81, 80, 71, 69, and 67 %, respectively (Additional file 1: Table S1). In this study, 5493/6144 (89.4 %) of PSQ reactions performed on culture- and smear-positive specimens and 500/1240 (40.3 %) of PSQ reactions performed on culture- and smear-negative specimens gave valid sequencing results. For smear-negative, culture-positive samples, 821/1160 reactions (70.3 %) provided useable sequence information, and for culture-negative, smear-positive samples, 234/464 (50.4 %) reactions provided valid sequencing results (Additional file 1: Table S2).

### Pyrosequencing (PSQ) indeterminate analysis

Results of the logistic regression analysis evaluating the variables associated with poor PSQ technical performance (PSQ indeterminate results) are displayed in Table 3. Multivariate logistic regression analysis showed that independent factors highly associated (adj OR > 2.0) with poor PSQ technical performance (indeterminate results) were: culture negativity (adj OR = 7.74), sequencing of either of two *rpoB* gene targets (adj OR = 5.29 and 4.65), sequencing of *gyrA* (adj OR = 4.07), sequencing in a Moldovan (adj OR = 2.86) site, sequencing of the *rrs* gene target (adj OR = 2.24), and AFB smear negativity (adj OR = 2.19). Sequencing in a South African site and sequencing of any gene target other than the IS*6110* marker were also significantly associated with increased odds of sequencing failures, adjusting for other covariates, though at lower levels than for the other variables (adj OR < 2.0).

**Table 3:** Multivariate logistic regression model of variables associated with poor pyrosequencing technical performance

Variable	Crude OR	95 % CI	Adjusted OR	95 % CI
AFB Smear				
Negative	**5.30**	4.76–5.90	**2.19**	1.91–2.51
Positive	1.00		1.00	
Culture				
Negative	**8.44**	7.51–9.49	**7.74**	6.67–8.99
Positive	1.00		1.00	
Site				
Moldova	**2.07**	1.84–2.34	**2.86**	2.47–3.31
South Africa	**1.86**	1.64–2.10	**1.50**	1.29–1.74
India	1.00		1.00	
Target				
*katG*	**1.52**	1.19–1.92	**1.69**	1.29–2.21
*inhA*	**1.30**	1.02–1.66	**1.38**	1.05–1.81
*ahpC*	**1.71**	1.35–2.16	**1.96**	1.50–2.55
*rpoB1*	**3.65**	2.93–4.55	**5.29**	4.12–6.79
*rpoB2*	**3.31**	2.65–4.12	**4.65**	3.62–5.97
*gyrA*	**2.99**	2.39–3.73	**4.07**	3.16–5.23
*rrs*	**1.90**	1.50–2.39	**2.24**	1.72–2.91
IS*6110*	1.00		1.00	

*p* < 0.01 for all bolded estimates except inhA (*p* < 0.05)

The number and type of errors observed for each PSQ gene target are shown in Table 4. No read errors were the most commonly occurring error for any gene target (49-91 % of all indeterminate PSQ reactions). The *gyrA* gene target had the highest percentage of no read errors (91 %) of any gene target. Homopolymer errors were a common cause of PSQ indeterminate results for the *katG* target (40 % of all indeterminate reactions). Instrument errors, mixed populations, and new mutations made up a minority of PSQ indeterminate calls for any gene target.

**Table 4:** Indeterminate pyrosequencing results: number and type of errors by gene target

TARGET	No Read Error	Homopolymer Error	Instrument Error	Other Error	Mixed Population	New Mutation	TOTAL	Error Rate (/1128 reactions)
IS*6110*	112 (85 %)	12 (9 %)	4 (3 %)	4 (3 %)	-	-	132	11.7 %
*katG*	91 (49 %)	74 (40 %)	1 (1 %)	19 (10 %)	1 (1 %)	-	186	16.5 %
*inhA*	121 (73 %)	9 (5 %)	3 (2 %)	29 (17 %)	2 (1 %)	2 (1 %)	166	14.7 %
*ahpC*	182 (87 %)	13 (6 %)	6 (3 %)	9 (4 %)	-	-	210	18.6 %
*rpoB1*	221 (61 %)	76 (21 %)	15 (4 %)	45 (12 %)	1 (0 %)	3 (1 %)	361	32.0 %
*rpoB2*	269 (78 %)	40 (12 %)	7 (2 %)	28 (8 %)	-	1 (0 %)	345	30.6 %
*gyrA*	294 (91 %)	2 (1 %)	6 (2 %)	18 (6 %)	1 (0 %)	1 (0 %)	322	28.5 %
*rrs*	149 (66 %)	30 (13 %)	-	48 (21 %)	-	-	227	20.1 %

## Discussion

Our investigation into the diagnostic and technical performance of PSQ in the GCDD study demonstrated the following: 1) The PSQ assay showed differences in diagnostic performance between clinical sites, especially with regards to the sensitivity of the assay in detecting KAN resistance in Moldova, 2) As an open sequencing platform, new gene targets may be added to the PSQ assay to improve diagnostic performance and accommodate our evolving knowledge of the molecular basis of TB drug resistance, and 3) The current PSQ assay protocols may be further improved by optimizing primers and PCR and sequencing parameters for each gene target included in the assay in order to decrease the number of indeterminate PSQ results. As this PSQ assay has great potential to curb the spread of M/XDR-TB, and its performance has recently been validated in a large multisite study, these results have important implications for future assay use and performance in diverse clinical environments while highlighting key areas for assay optimization.

### Pyrosequencing diagnostic performance between sites

Differences in diagnostic sensitivity were noted for various drug compounds between the three clinical sites. South Africa showed lower sensitivity for the detection of INH resistance (71 %, 95 % CI 0.53-0.85) than India or Moldova, as the PSQ assay did not detect resistance-associated mutations in 25 of 35 phenotypically INH-resistant specimens evaluated in South Africa. This result suggests that these strains do not to have the expected *katG*, *inhA*, and/or *ahpC* mutations found in approximately 94 % of INH-resistant strains, globally [22]. One reason for this discordance might be the failure of our assay to include additional gene regions associated with INH resistance, such mutations in the *fab*G1 gene or outside regions of *katG* [23, 24], including *katG* mutations at codons 139, 142, 269, 385, 387 and 541, recently associated with high INH minimum inhibitory concentrations (>10 μg/mL) [24]. If any of these mutations are common in the South African population enrolled in our study, then it may be worthwhile to incorporate one or more of these gene regions into the next version of our PSQ assay. However, as only 35 phenotypically INH-resistant South African specimens were available for analysis, and the confidence intervals for the calculation included values as high as 85 %, the low sensitivity estimate we observed may also have been an artifact of small sample size.

The PSQ assay also demonstrated lower sensitivity for the detection of RIF resistance in South Africa (77 %, 95 % CI 0.54–0.91) than in India. The assay did not detect mutations in five of the 22 phenotypically RIF-resistant samples evaluated in South Africa. As it is unlikely that these specimens lacked the resistance-associated *rpoB* mutations found in approximately 96 % of all RIF-resistant strains [22], this result was also likely due to the small sample size of phenotypically RIF-resistant South African samples available for analysis, as the confidence intervals for this calculation included values as high as 91 %. However, it might also be worth investigating *rpoB* gene regions outside of those evaluated in this study, to ensure that no rare mutations are present in these samples in future studies.

For the detection of FQ resistance, PSQ demonstrated lower sensitivity in Moldova (64–67 %, 95 % CI 0.36–0.87) than in India. As 93 % of all FQ-resistant strains have mutations in the *gyrA* gene region included in our assay [22], this result was lower than expected. However, our diagnostic sensitivity measures were in the range of those reported by Lacoma et al., who reported 40 % sensitivity for detection of OFX resistance and 70.8 % for the detection of MOX resistance for a PSQ assay including the same *gyrA* gene regions as our study, tested against strains from Spain and Lithuania [25]. Furthermore, only 14–15 phenotypically MOX- and OFX-resistant specimens were analyzed in Moldova, which may have led to a chance oversampling of specimens missing these common mutations. This possibility is reflected in the upper limits of the confidence intervals for this estimate, which include values as high as 87 %. As with the detection of phenotypic INH and RIF resistance, although the point estimates for the sensitivity of the detection for phenotypic FQ resistance were lower in one clinical site, no significant differences could be confirmed based upon the spread of the confidence intervals surrounding those sensitivity estimates.

For the detection of injectable resistance, our PSQ assay showed lower sensitivity in Moldova than in India for all drugs. The sensitivity of the assay for the detection of CAP resistance in Moldova (40 %, 95 % CI 0.14–0.73) was lower than expected, as only four of the 10 phenotypically CAP-resistant specimens evaluated in this site were found to have the expected *rrs* 1401G mutation, previously documented to occur in 88 % of CAP-resistant specimens, globally [22]. For the detection of AMK resistance in Moldova, the assay also demonstrated lower sensitivity (33 %, 95 % CI 0.11–0.65) than expected, as only four of 12 phenotypically AMK-resistant specimens were determined to have the *rrs* 1401G mutation found in approximately 84 % of all AMK-resistant specimens, globally [22]. Although these discordances are likely related to the small sample size of AMK- and CAP-resistant specimens evaluated in Moldova, these observed discordances might also result from the failure of our assay to include additional gene regions associated with injectable resistance, such as the *rrs* 1484T mutation or *tlyA* mutations [26]. Indeed, other studies of tests relying upon the *rrs* 1401G mutation for AMK and CAP resistance detection have reported sensitivities as low as 57 % [27], and so this result may be worth further investigation. There is also the possibility that *rrs* 1401 or 1402 mutations were present in the specimens, but were missed by our PSQ assay for some reason, which would call for a closer look into the ability of PSQ to accurately sequence this gene region. These specimens are currently being further evaluated by whole genome sequencing to identify the molecular basis of phenotypic injectable resistance. By far, however, the most notable difference in observed sensitivity for any injectable between the sites was for the detection of KAN resistance in Moldova, where only 7 % (95 % CI 0.02–0.18) of the 57 phenotypically KAN-resistant specimens were found to have the *rrs* 1401G mutation, versus 89 % in India and 92 % in South Africa.

### Diagnostic performance following *eis* promoter addition

In Moldova, a high number of specimens showed resistance to KAN but not to the other injectable compounds (AMK and CAP). This fact is unsurprising, as kanamycin was widely used for TB treatment in the former Soviet Union, selecting for resistance to this compound [28, 29]. The high number of KAN-resistant *Mtb* clinical specimens without *rrs* mutations in Moldova (*n* = 53) suggested that other genes or gene regions were involved in conferring KAN resistance in this site. Upon the addition of *eis* promoter sequencing capability to the PSQ assay, a dramatic change in sensitivity for KAN resistance detection was observed in Moldova (7 % to 79 %), confirming the role of *eis* promoter mutations in conferring KAN resistance in this population. Notably, the addition of the gene region to the assay in India also resulted in a sensitivity improvement for the detection of KAN resistance (89 % to 93 %). However, the improved sensitivity came at a loss to assay specificity in both sites for KAN resistance detection, due to the presence of *eis* promoter mutations in KAN-susceptible specimens. In order to comment upon this discrepancy, 15 KAN-susceptible Indian specimens confirmed to have *eis* promoter mutations were subjected to repeat phenotypic KAN DST at the critical concentration (2.5 μg/mL). All DST reactions were run in duplicate. Eleven of the 15 specimens (73 %) showed a resistant phenotype in at least one of the two duplicate DST runs, but five of these results were mixed (one run being susceptible, the other resistant). Four discrepant specimens were KAN susceptible in both DST runs. These results underscore the fact that mutations in this gene region should to be studied further to quantitate their association with phenotypic KAN resistance, especially as these mutations confer only low-level KAN resistance, which may or may not be picked up by phenotypic DST at just one critical concentration (2.5 μg/mL, in our study) [30]. Although these results suggest a reexamination of the critical concentration to establish KAN phenotypic resistance, the addition of this gene region into our assay confirms the adaptability of our molecular diagnostic platform for diverse clinical environments.

### Pyrosequencing (PSQ) technical performance across all sites

A current limitation of PSQ as an M/XDR-TB diagnostic is its high rate of sequencing failure. In our study, 25 % of all sequencing reactions failed to generate an interpretable sequencing read [11], with results varying by specimen smear- and culture-status. The variable most highly associated with poor PSQ technical performance was culture negativity, over AFB smear negativity. This difference is unsurprising as culture is a more sensitive test for *Mtb* compared to AFB smear. However, over 40 % of sequencing reactions conducted on smear- and culture-negative samples still yielded sequencing results. Although culture-negative samples are generally considered to be samples in which the *Mtb* bacteria is not present, it is likely that our assay was indeed detecting *Mtb* DNA, as the primers designed for our assay are highly specific for *Mtb*. This DNA may have come from dead *Mtb* bacteria present in the samples, which is likely seen when processing samples from patients previously treated for TB infections, and so this finding emphasizes the importance of DR-TB diagnostic results interpretation in the context of patient clinical presentation and TB treatment history*.*

The ability of our assay to sequence a large portion of AFB smear-negative *Mtb* clinical specimens underscores the utility of this molecular diagnostic for a diverse range of clinical samples. Indeed, our 70.8 % sequencing success for smear-negative, culture-positive specimens is better than reported for the GeneXpert assay (55 %) [31]. As many laboratories lack the sterile conditions or equipment necessary to perform AFB smear, culture and DST of *Mtb*, and a large portion of TB infections remain smear-negative despite clinical and radiological signs of disease, PSQ presents a valid alternative to conventional growth-based diagnostic methods [32]. Although the presence of *Mtb* DNA does not necessarily confirm the presence of viable bacteria in a sample (as with culture-negative samples), PSQ can potentially provide the clinician with information about a portion of smear-negative infections when a diagnosis is otherwise elusive, as long as the results are considered in the context of the patient’s clinical presentation and past and current TB treatment regimens [33].

In addition to culture and smear result, the proportion of interpretable sequencing results in our study appeared to vary significantly by gene. Interestingly, after culture negativity, sequencing of the *rpoB* gene target in either one of two sequencing reactions was the variable with the highest adjusted odds of sequencing failure. Poor *rpoB* sequencing success is likely a result of the higher order DNA structures present in the *Mtb* genomic DNA at this gene region, preventing DNA access and therefore resulting in PCR and/or sequencing failure [34]. Although these higher order DNA structures are inherent to the *rpoB* gene, their presence might be addressed by altering PCR and/or sequencing reaction conditions, such as increasing the melting and extension temperatures during PCR or introducing reaction additives to prevent the formation of such structures. Additionally, the two *rpoB* sequencing products were the longest in this study. This factor appeared to contribute to the occurrence of indeterminate results by increasing the number of unresolvable homopolymer errors seen in these reactions [23]. Increasing the number of sequencing reactions for a given target, thereby shortening the length of the sequencing products, may rectify any issues related to gene target sequencing length such as seen with the *rpoB* gene targets. Sequencing of the *gyrA* target had the next highest odds of indeterminate results. The high number of indeterminate results seen for *gyrA* target sequencing reactions appeared to result from amplification errors, or primer hybridization during PCR, as the majority of *gyrA* sequencing errors were characterized as no read errors. Like *rpoB*, *gyrA* might form stable secondary DNA structures at the PCR temperatures and conditions used for our assay. This factor may be addressed in future versions of the PSQ assay by redesigning the *gyrA* PCR primers for this reaction or by adjusting PCR temperatures to relax these higher-order structures. The variable with the next highest odds of sequencing failure was sequencing in Moldova. As the gene regions sequenced by the PSQ assay are highly conserved among TB strains worldwide [35, 36], it is highly unlikely that this poor technical performance observed in Moldova is related to the genetics of the TB specimens in this site. Instead, this association is likely tied to methodological factors affecting PCR and PSQ in Moldova versus other sites, such as long delays between DNA extraction, PCR and sequencing. In our study, the Moldovan site was known to batch *Mtb* samples more than the other two sites, performing PSQ only once a week- a factor that may explain technical performance differences between the sites. A final gene target highly associated with indeterminate results was the *rrs* gene. The majority of *rrs* indeterminate results were classified as no read errors, indicating potential for PCR and sequencing optimization, similar to the *gyrA* target. This analysis highlights important areas for assay technical performance improvement, and many of these problematic gene targets may be easily optimized in future versions of the assay.

## Conclusions

Although our PSQ assay was generally a high performing M/XDR-TB diagnostic across three diverse clinical environments, notable reductions in sensitivity were identified between the three sites, especially for KAN resistance detection in Moldova. The flexibility of the PSQ assay allowed us to quickly update the platform when this performance lapse was identified, improving assay sensitivity for KAN resistance detection. Additionally, we found the PSQ assay to generate data for a large proportion of smear-negative samples, comparable to GeneXpert, and our analysis of the additional variables associated with poor PSQ technical performance highlighted gene targets for optimization to further improve the assay’s technical performance. These results have important implications for the use and interpretation of PSQ assays as M/XDR-TB diagnostics, and may serve to inform other molecular M/XDR-TB diagnostics that interrogate similar gene targets in clinics across the globe.

## Additional files

Additional file 1: Table S1. Pyrosequencing (PSQ) Success of Each Gene Target by Smear- and Culture-Status. **Table S2.** Proportion of Successful Pyrosequencing (PSQ) Reactions by Smear- and Culture-Status. (DOCX 18 kb) Additional file 2: Pyrosequencing (PSQ) Performance Dataset. (TXT 400 kb) Additional file 3: Pyrosequencing (PSQ) Technical Dataset. (CSV 303 kb)

## Abbreviations

AFB: Acid-fast bacilli
AMK: Amikacin
CAP: Capreomycin
DR-TB: Drug-resistant TB
DST: Drug susceptibility testing
FQs: Fluoroquinolones
GCDD: Global consortium for drug-resistant TB diagnostics
INH: Isoniazid
KAN: Kanamycin
MDR-TB: Multidrug-resistant tuberculosis
MGIT: Mycobacterial growth indicator tube
MOX: Moxifloxacin
*Mtb*: *Mycobacterium tuberculosis*
OFX: Ofloxacin
PSQ: Pyrosequencing
RIF: Rifampicin
TB: Tuberculosis
XDR-TB: Extremely drug-resistant tuberculosis
