# Supplementary material for: Shedding light on the performance of a pyrosequencing assay for drug-resistant tuberculosis diagnosis
Source: BMC Infect Dis. 2016 Aug 31;16(1):458. doi: 10.1186/s12879-016-1781-y (PMC5006534; doi:10.1186/s12879-016-1781-y)
Supplement: Additional file 1: Table S1. — Pyrosequencing (PSQ) Success of Each Gene Target by Smear- and Culture-Status. Table S2. Proportion of Successful Pyrosequencing (PSQ) Reactions by Smear- and Culture-Status. (DOCX 18 kb) [file 12879_2016_1781_MOESM1_ESM.docx]

**Table S1.** Pyrosequencing (PSQ) Success of Each Gene Target by Smear- and Culture-Status.

| Sequencing Result | | Culture Negative (n=213) | | Culture Positive (n=914) | | Culture I*  (n=1) | | Total n (%) |
| --- | --- | --- | --- | --- | --- | --- | --- | --- |
|  |  | Smear Positive | Smear Negative | Smear Positive | Smear Negative | Smear Positive | Smear Negative |  |
| IS*6110* | Positive | 37 | 91 | 738 | 127 | 0 | 1 | 994 (88) |
|  | Indeterminate | 21 | 64 | 30 | 18 | 0 | 0 | 133 |
| *katG* | Positive | 40 | 90 | 690 | 116 | 0 | 1 | 937 (83) |
|  | Indeterminate | 18 | 65 | 78 | 29 | 0 | 0 | 190 |
| *inhA* | Positive | 38 | 85 | 717 | 120 | 0 | 0 | 960 (85) |
|  | Indeterminate | 20 | 70 | 51 | 25 | 0 | 1 | 167 |
| *ahpC* | Positive | 25 | 58 | 721 | 113 | 0 | 0 | 917 (81) |
|  | Indeterminate | 33 | 97 | 47 | 32 | 0 | 1 | 210 |
| *gyrA* | Positive | 23 | 33 | 667 | 82 | 0 | 0 | 805 (71) |
|  | Indeterminate | 35 | 122 | 101 | 63 | 0 | 1 | 322 |
| *rrs* | Positive | 30 | 63 | 712 | 94 | 0 | 0 | 899 (80) |
|  | Indeterminate | 28 | 92 | 56 | 51 | 0 | 1 | 228 |
| *rpoB1* | Positive | 22 | 40 | 613 | 82 | 0 | 0 | 757 (67) |
|  | Indeterminate | 36 | 115 | 155 | 63 | 0 | 1 | 370 |
| *rpoB2* | Positive | 19 | 40 | 635 | 87 | 0 | 0 | 781 (69) |
|  | Indeterminate | 39 | 115 | 133 | 58 | 0 | 1 | 346 |
|  | Total | 464 | 1240 | 6144 | 1160 | 0 | 8 | 9016 |

*I, indeterminate. Culture was contaminated.

**Table S2.** Proportion of Successful Pyrosequencing (PSQ) Reactions by Smear- and Culture-Status.

|  |  | CULTURE | |
| --- | --- | --- | --- |
|  |  | Positive (n=7312) | Negative (n= 1704) |
| SMEAR | Positive (n=6608) | 89.4%  (5493/6144) | 50.4%  (234/464) |
|  | Negative (n= 2416) | 70.3%  (821/1168) | 40.3%  (500/1240) |

One culture-indeterminate sample was excluded from analysis in the above table.
